# Ribosome footprint profiling enables elucidating the systemic regulation of fatty acid accumulation in *Acer truncatum*

**DOI:** 10.1186/s12915-023-01564-8

**Published:** 2023-04-03

**Authors:** Qiuyue Ma, Yuxiao Wang, Shushun Li, Jing Wen, Lu Zhu, Kunyuan Yan, Yiming Du, Shuxian Li, Liping Yan, Zhijun Xie, Yunzhou Lyu, Fei Shen, Qianzhong Li

**Affiliations:** 1 Institute of Leisure Agriculture, Jiangsu Academy of Agricultural Sciences, Jiangsu Key Laboratory for Horticultural Crop Genetic Improvement Nanjing, Nanjing, 210014 China; 2 Nanjing Forestry University, Nanjing, 210037 China; 3 Shandong Academy of Forestry Sciences, Jinan, 250014 China; 4 Xiangyang Forestry Science and Technology Extension Station, Xiangyang, 441000 China; 5 Jiangsu Academy of Forestry, Nanjing, 211153 China; 6 Institute of Biology, Beijing Academy of Agriculture and Forestry Sciences, Beijing, 100197 China

**Keywords:** *Acer truncatum*, Ribosome profiling, Transcriptome

## Abstract

**Background:**

The accumulation of fatty acids in plants covers a wide range of functions in plant physiology and thereby affects adaptations and characteristics of species. As the famous woody oilseed crop, *Acer truncatum* accumulates unsaturated fatty acids and could serve as the model to understand the regulation and trait formation in oil-accumulation crops. Here, we performed Ribosome footprint profiling combing with a multi-omics strategy towards vital time points during seed development, and finally constructed systematic profiling from transcription to proteomes. Additionally, we characterized the small open reading frames (ORFs) and revealed that the translational efficiencies of focused genes were highly influenced by their sequence features.

**Results:**

The comprehensive multi-omics analysis of lipid metabolism was conducted in *A. truncatum.* We applied the Ribo-seq and RNA-seq techniques, and the analyses of transcriptional and translational profiles of seeds collected at 85 and 115 DAF were compared. Key members of biosynthesis-related structural genes *(LACS*, *FAD2*, *FAD3*, and *KCS*) were characterized fully. More meaningfully, the regulators (*MYB*, *ABI*, *bZIP*, and *Dof*) were identified and revealed to affect lipid biosynthesis via post-translational regulations. The translational features results showed that translation efficiency tended to be lower for the genes with a translated uORF than for the genes with a non-translated uORF. They provide new insights into the global mechanisms underlying the developmental regulation of lipid metabolism.

**Conclusions:**

We performed Ribosome footprint profiling combing with a multi-omics strategy in *A. truncatum* seed development, which provides an example of the use of Ribosome footprint profiling in deciphering the complex regulation network and will be useful for elucidating the metabolism of *A. truncatum* seed oil and the regulatory mechanisms.

**Supplementary Information:**

The online version contains supplementary material available at 10.1186/s12915-023-01564-8.

## Background

Woody plants and oil crops are important resources in China. Accelerating the development and production of woody plants and oil crops is important for ensuring food and oil security. *Acer truncatum* is a versatile oil-producing woody tree and is a valuable ornamental species [1, 2]. In 2011, seed oil was approved as a New Resource Food by the National Health and Family Planning Commission of the People’s Republic of China, with important implications for various industries (e.g., food and medicine). *Acer truncatum* seed oil is composed mainly of triacylglycerols (TAGs), with approximately 90% unsaturated fatty acids, including oleic acid (C18:1), linoleic acid (C18:2), α-linolenic acid (C18:3), and nervonic acid (C24:1) [3–5]. The proportions of oleic acid and linoleic acid are higher than those in peanut (*Arachis hypogaea*), sunflower (*Helianthus annuus*), and rapeseed (*Brassica napus*) oils [6]. Additionally, nervonic acid (24:15, cis-15-tetracosenoic acid, n-9) is a rare fatty acid (FA) accounting for 5–6% of the *A. truncatum* seed oil, making it potentially useful for treating schizophrenia, psychosis, and attention deficit disorder [7, 8].

The complex biochemical reactions mediating lipid biosynthesis have been studied in many plants [4, 9–11], but the underlying regulatory mechanisms remain relatively uncharacterized. In plants, the general lipid biosynthesis pathway includes the following four steps: de novo FA synthesis in plastids, acyl elongation and editing, TAG assembly in the endoplasmic reticulum, and oil drop formation [9, 12]. Some studies demonstrated that ACCase and 3-ketoacyl CoA synthetase (KCS) are the key rate-determining enzymes controlling the synthesis of FAs and very long-chain monounsaturated fatty acids (VLCFAs), respectively [5, 13]. Triacylglycerols are stored in oil bodies surrounded by a lipid monolayer. Moreover, previous research indicated that TAG assembly is critical for modulating lipid accumulation in developing oilseeds [14, 15]. The molecular basis of lipid biosynthesis and the underlying regulatory mechanism has been studied in terms of the associated genes (i.e., transcription) and proteins (e.g., enzymes) in different plants [13, 16, 17]. Changes in the abundance of the candidate enzymes involved in lipid biosynthesis are not correlated with changes in the expression of the cognate mRNAs [10]. Post-transcriptional regulation affects the production of enzymes catalyzing lipid biosynthesis-related reactions, but there is relatively little available information regarding the post-transcriptional control of lipid biosynthesis in *A. truncatum*.

Ribosome footprinting (Ribo-seq) is a recently developed high-throughput sequencing technique that can generate precise information about translation at the genome-wide level and a single-codon resolution [18, 19]. In a Ribo-seq experiment, ribosomes are immobilized and the lysate is treated with nucleases to obtain ribosome-protected mRNA fragments (ribosome footprints). Then the deep sequencing of the ribosome footprints allowed for comprehensive and precise mapping of the positions of translating ribosomes on transcripts, which could reveal the quantity and interest positions of ribosomes [20, 21]. Thus, it has been used to clarify the translational regulation of specific tissues in diverse species, including *Arabidopsis thaliana* [22], maize [23], and humans [24]. Additionally, global three-nucleotide periodicity and translational efficiency (TE) revealed by ribosome profiling also provide valuable information for identifying novel open reading frames (ORFs) and ribosomal pause sites as well as for determining elongation [25].

## Results

### Establishment of an experimental and data analysis pipeline for the *A. truncatum* translatome

**Fig. 1:**
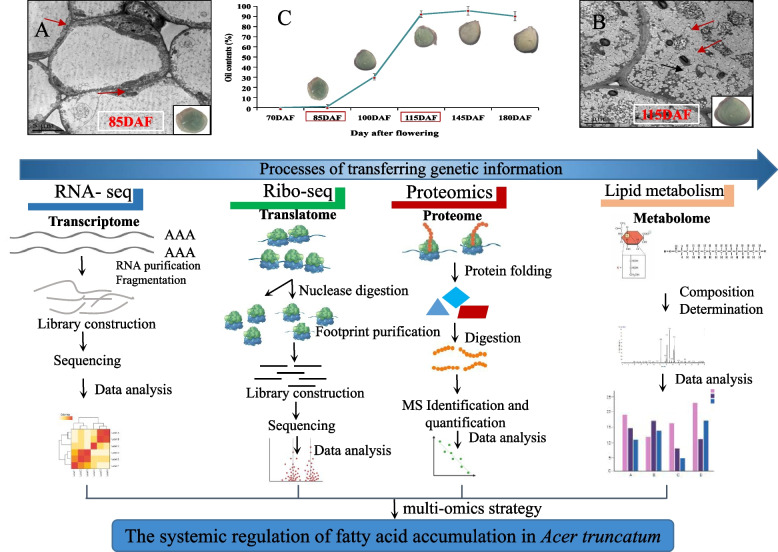
Overview of the experimental design. **A**, **B** Electron microscopy images of oil bodies in seed cells at 85 and 115 DAF are presented. The red arrow represents the oil body. **C** The seed development of *A. truncatum*

In a previous study, we found that the oil proportions rapidly increased from 85 to 115 DAF, while the content variation of the components from 115 to 180 DAF was not significant [5]. in this study, the electron microscopy images of oil bodies were also conducted in seed cells at 85 and 115 DAF, the results showed that no lipid bodies were found at 85 DAF, and the lipid bodies filled the endosperm cells in 115 DAF (Fig. 1C). Therefore, the period between 85 and 115 DAF is critical for *A. truncatum* oil production. To investigate genome-wide gene expression related to the regulation of seed development, the ribosomal profiles of the seeds collected at these two time points were analyzed based on Ribo-seq, RNA-seq, and proteomic data. The experimental strategy is presented in Fig. 1A–C. The DEGs of 115 DAF/85 DAF were identified at transcriptional, translational, and protein levels. Then we performed the KEGG analysis to identify the lipid-enriched pathways among the up-regulated key DEGs, to know more the molecular basis of *A. truncatum* seed oil production. Finally, we combined Ribo-seq and RNA-seq techniques, enabled the identification of ORFs in *A. truncatum* seeds, and revealed their translational features. For the seeds collected at 85 and 115 DAF, we obtained 569.3–667.5 million and 614.3–706.6 million clean reads from RNA-seq libraries, respectively. Meanwhile, the 5.8–7.1 million and 5.7–6.5 million clean reads were generated from the Ribo-seq libraries, respectively (Table S2). A total of 31,738 peptides were obtained and 7,039 proteins were identified during the proteomic analysis. Details (e.g., identities and quantities) regarding the transcriptome, translatome, and proteome analyses are provided in Tables S3–5. The principal component analysis of the transcriptome, translatome, and proteome data clearly distinguished the seeds collected at 85 DAF from the seeds collected at 115 DAF (Fig. S1). In the present study, the seeds collected at 115 DAF contained 2.65 g/100 g oleic acid, 3.25 g/100 g linoleic acid, and 0.69 g/100 g nervonic acid, which were respectively 3.04-fold, 4.58-fold higher, and 13.8-fold than the corresponding contents in the seeds collected at 85 DAF (Table S6 and Fig. S2). These results reflected the reproducibility and the clear differences between the seeds collected at 85 and 115 DAF at the transcription, translation, and protein levels.

### Features of ribosomal footprints

**Fig. 2:**
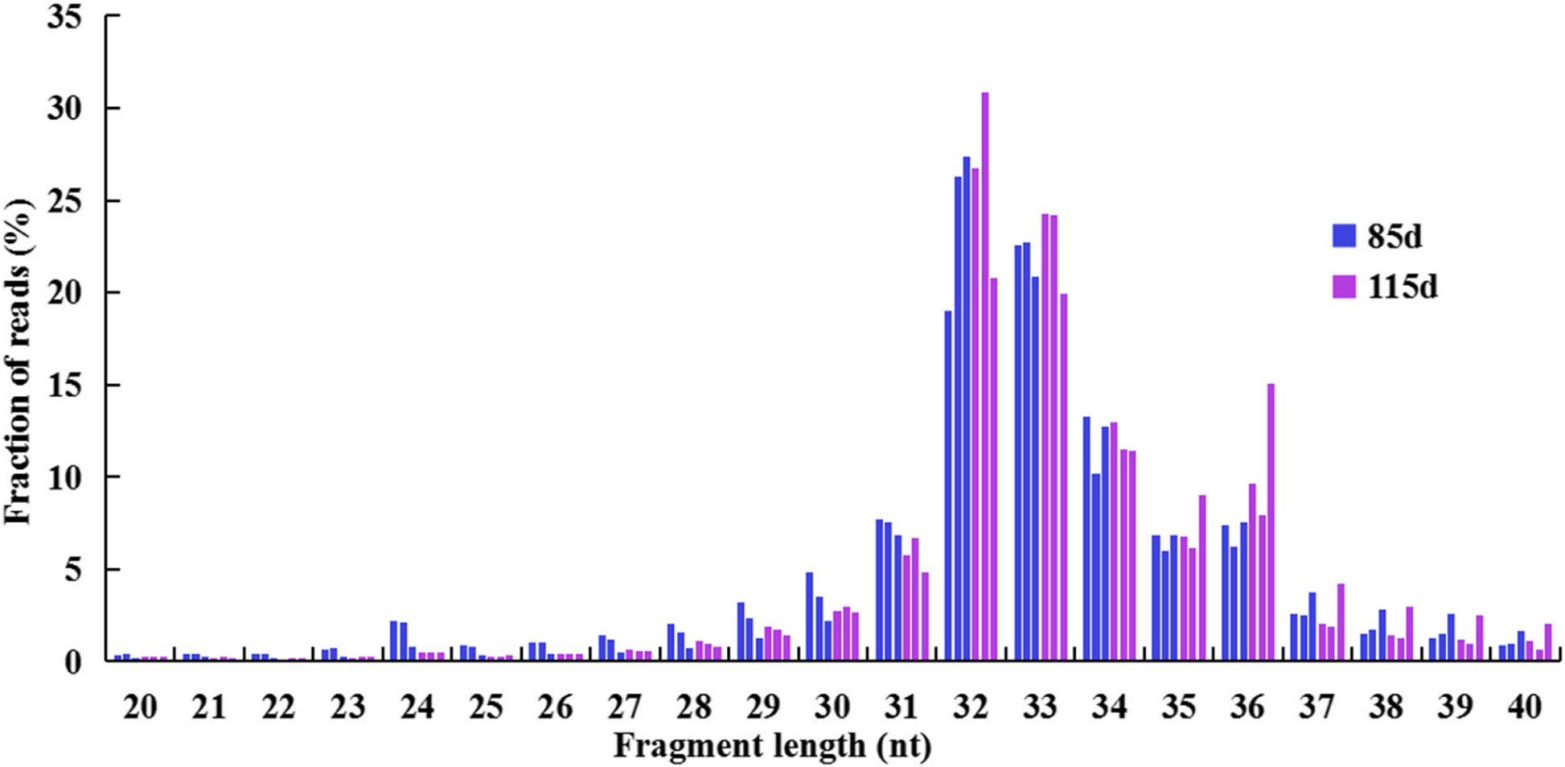
Characteristics of ribosome profiling data in 85 and 115 DAF seeds. Length distribution of RPFs in 85 and 115 DAF seeds. The same color bars refer to three biological replicates

**Fig. 3:**
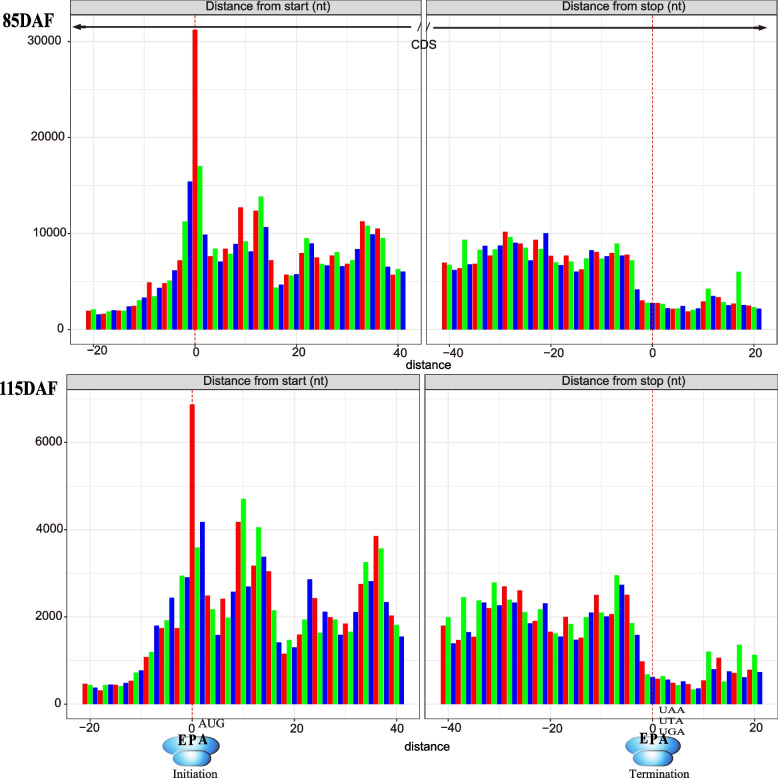
The total number of RPFs along the start and stop codon regions in 85 and 115 DAF of *A. truncatum* seeds. The red, green, and blue bars represent the first, second, and third reading frames, respectively

High-quality ribosomal footprints are critical for detecting ORFs. To explore whether the ribosomal footprint features change as *A. truncatum* seeds develop, the basic ribosomal profiles of the RPFs were compared between the seeds collected at 85 and 115 DAF. Our findings were consistent with the reported RPF length distributions in *A. thaliana* and maize [22, 26]. The predominant RPF length was 32–33 nt for the *A. truncatum* seeds (Fig. 2). In our study, the three-nucleotide periodicity was detected around the start and stop codon regions of the RPFs with differing read lengths (Fig. 3). Moreover, 86.94% and 88.55% of the RPFs for the seeds collected at 85 and 115 DAF, respectively, were located in the coding sequence (CDS) regions, whereas less than 6% of the RPFs were detected in the other regions (Fig. 3). Similar results were reported for other plant species [26–28]. Previous studies confirmed the importance of 5′ and 3′ UTRs for the post-transcriptional regulation of gene expression [29, 30]. Additionally, the overall ribosome binding profiles were similar; the robustness of the three-nucleotide periodicity was quantified based on the percentage of reads in the expected reading frame. Considered together, these results indicated high-quality Ribo-seq data were obtained in this study.

### Multi-omics-based functional comparison and analysis of the seeds at critical time points

**Fig. 4:**
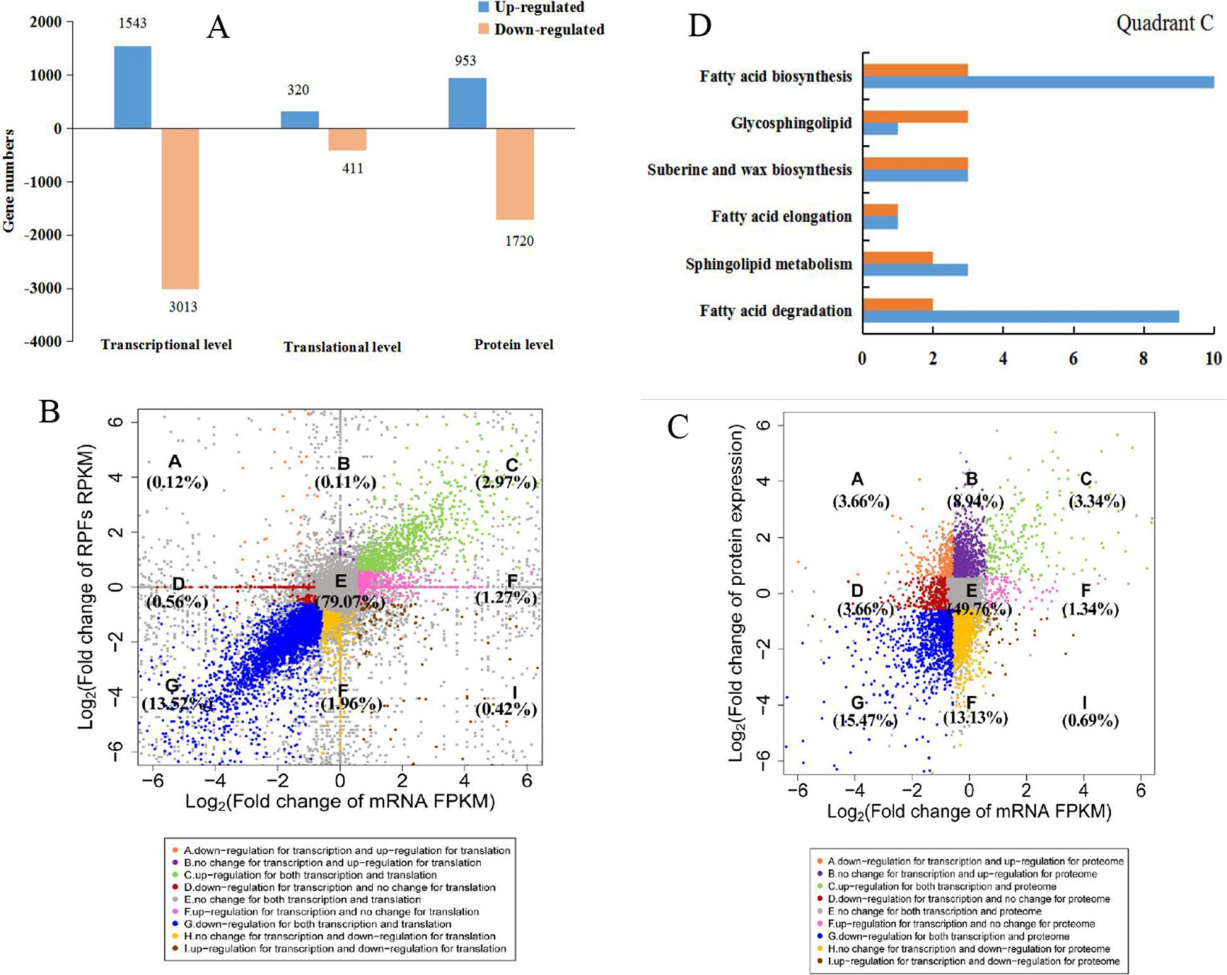
Transcriptional, translation, and protein regulation of 85 and 115 DAF seeds in *A. truncatum*. **A** The number of DEGs at transcriptional or translational levels, the blue and orange bars refer to the number of up-regulation and down-regulated genes, respectively. **B** Scatter plot of the fold change of 115 DAF/85 DAF group at transcriptional and translational levels. **C** Scatter plot of the fold change of 115 DAF/85 DAF group at transcriptional and protein levels. **D** KEGG enrichment analysis of lipid genes in quadrants C, up-regulation for transcriptional and translational (orange column), up-regulation for both transcriptional and protein (blue column)

To further characterize the transcription, translation, and protein level differences between *A. truncatum* seeds at different stages of development, we applied a multi-omics approach. A differential expression analysis was conducted to characterize the changes in global gene expression in the transcription, translation, and protein levels, respectively. A total of 1543 up-regulated and 3013 down-regulated DEGs (transcription level), 320 up-regulated and 411 down-regulated DEGs (translation level), and 953 up-regulated and 1720 down-regulated DRPs (protein level) in 115 DAF vs. 85 DAF. (Fig. 4A). To clarify the overall tendencies in the gene expression changes, the scatter plots of the fold-changes in the transcription and translation levels (Fig. 4B) and the transcription and protein levels (Fig. 4C) were constructed by the comparison of the seeds collected at 115 DAF vs. 85 DAF. We subdivided all of the DEGs into nine categories (|log_2_[fold-change in FPKM]|≥ 1 and FDR < 0.05). Quadrant E included the genes with non-significant changes in expression at the transcription and translation levels (79.07%) as well as at the transcript and protein levels (49.76%). Quadrant C comprised the genes that were up-regulated at the transcription and translation levels (2.97%) as well as at the transcript and protein levels (3.34%). Details regarding the genes in different quadrants are listed in Tables S7–8. To elucidate the biological functions of the DEGs between the seeds collected at the two time points, we also performed a KEGG analysis to identify the enriched pathways among the genes. The genes that were translated at higher levels in the seeds collected at 115 DAF than in the seeds collected at 85 DAF were in quadrant C (Fig. 4D). The examination of the transcription and translation levels revealed 20 DEGs associated with FA metabolism and three DEGs associated with suberine and wax biosynthesis. The analysis of the transcript and protein levels detected seven and three DEGs associated with FA metabolism and suberine and wax biosynthesis, respectively. These DEGs included *LACS* and *KCS* genes (Tables S7–8). This was consistent with the observation that the FA proportions rapidly increased from 85 to 115 DAF. In addition, we also found 151 up-regulated and 362 down-regulated DEGs about translational efficiency in 115 DAF vs. 85 DAF (FDR < 0.05 and absolute fold-change ≥ 1.5) (Fig. S3A). The KEGG analysis results showed that six differential translational efficiency genes enriched in lipid metabolism (Fig. S3B and Table S9). We speculated that they might play an important role in the transcription and translation of lipid biosynthesis. Our findings regarding the FA metabolism at these two-time points may provide researchers with important insights into the accumulation of oil and the mechanism regulating FA synthesis in *A. truncatum* seeds.

### Insights into lipid biosynthesis derived from a multi-omics analysis

The morphological characteristics of developing seeds at five time points are presented in Fig. 1C. From 85 to 115 DAF, the lipid bodies gradually increased in size and eventually filled the endosperm cells (Fig. 1A and B). Moreover, the analysis of the oil content and composition detected the following eight dominant components: palmitic acid (C16:0), stearic acid (C18:0), oleic acid (C18:1), linoleic acid (C18:2), linolenic acid (C18:3), eicosenoic acid (C20:1), erucic acid (C22:1), and nervonic acid (C24:1) (Table S6 and Fig. 1). The oil content was much higher in the seeds collected at 115 DAF than in the seeds collected at 85 DAF. Furthermore, individual FAs were biosynthesized and accumulated at different rates during the development of *A. truncatum* seeds.

**Fig. 5:**
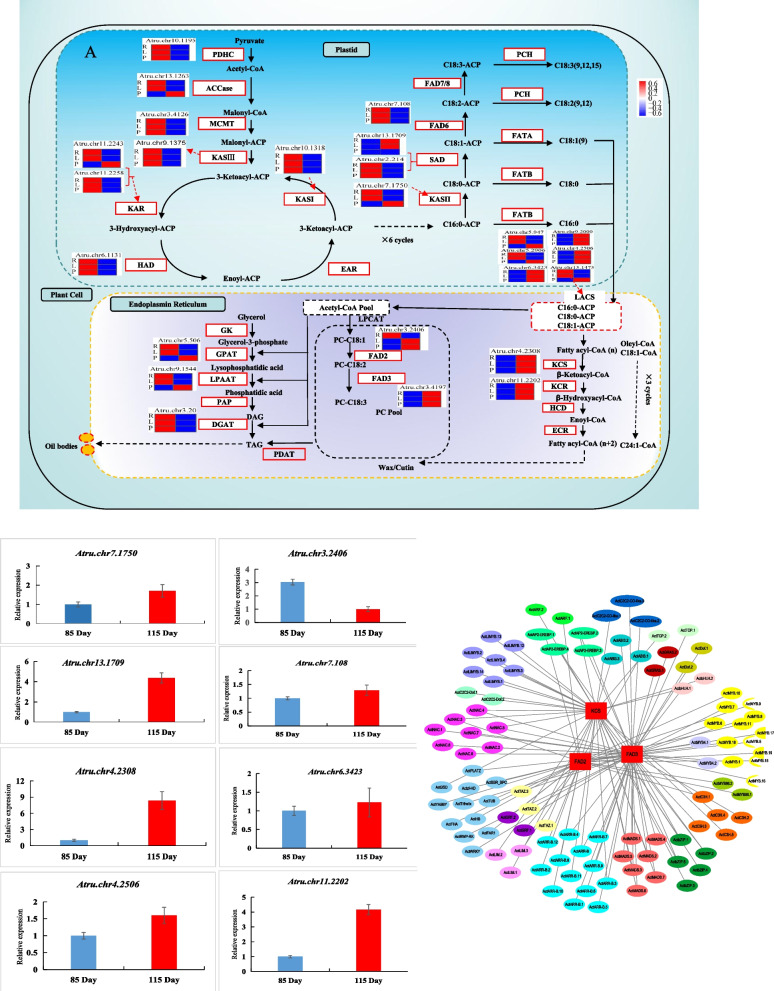
**A** Regulation on lipid biosynthesis-related pathways. The icons next to the key enzymes represent the relative expression levels of the transcripts at 85 and 115 DAF. The heat map is displayed according to log10 [fragments per kilobase per million (FPKM) + 1] values. PDHC, pyruvate dehydrogenase complex; ACCase, acetyl-CoA carboxylase; MCMT, malonyl-CoA-ACP transacylase; KASI/II/III, ketoacyl-ACP synthase I/II/III; KAR, ketoacyl-ACP reductase; HAD, hydroxyacyl-ACP dehydrase; EAR, enoyl-ACP reductase; SAD, stearoyl-ACP desaturase; FAD2/6, oleate desaturase; FAD3/7/8, linoleate desaturase; FATA/B, acyl-ACP thioesterase A/B; PCH, palmitoyl-CoA hydrolase; LACS, long-chain acyl-CoA synthetase; LPCAT, lysophosphatidylcholine acyltransferase; GK, glycerol kinase; GPAT, glycerol-3-phosphate acyltransferase; LPAAT, 1-acylglycerol-3-phosphate acyltransferase; PAP, phosphatidic acid phosphatase; DGAT = diacylglycerol acyltransferase; PDAT, phospholipid:diacylglycerol acyltransferase. **B** The qRT-PCR validation of oil biosynthesis genes at two-time points of *A. truncatum*. The relative expression levels of genes were normalized with the internal reference gene Actin (Wang et al., 2018). Three biological replicates (nested with three technical replicates) are represented by the error bars (*P* < 0.05). **C** TF-oil biosynthesis network. Co-expressed network of the correlation among transcription factors and oil biosynthesis genes (*FAD2*, *FAD3*, and *KCS*). The gray lines indicate the correlations between the two genes

To further investigate the major lipid biosynthesis pathway in the critical period for oil accumulation in *A. truncatum* seeds, we combined lipid biosynthesis-related pathways with heatmaps for the mRNA, RPF, and protein levels and constructed the oil biosynthesis pathway (Fig. 5A). The results of the KEGG pathway analysis indicated 25 genes identified in our multi-omics analysis encode key enzymes involved in FA biosynthesis; their expression levels were determined for the seeds collected at 85 and 115 DAF. Ten genes were selected for qRT-PCR analysis to validate the RNA-seq results. The qRT-PCR data for the analyzed genes were generally consistent with the corresponding RNA-seq data (Fig. 5B). In plants, FA production involves the de novo synthesis and elongation of FAs in plastids and the desaturation of FAs and TAG assembly in the endoplasmic reticulum [5, 31, 32]. However, there have been relatively few studies that examined FA synthesis based on a multi-omics approach (i.e., transcriptomic, translatomic, and proteomic). Fatty acids are synthesized by a series of reactions, after which LACS on the outer membrane of the plastid catalyzes the esterification of FAs to generate the acyl-CoA pool. In the current study, six homologous *LACS* genes were identified, among which *Atru.chr 9.2000*, *Atru.chr1.2506*, and *Atru.chr6.342* were highly expressed at the transcription, translation, and protein levels. The C18:1 and C18:2 FAs accounted for the largest proportion of the FAs in *A. truncatum* seeds. Previous studies demonstrated that the accumulation of C18:1 and C18:2 FAs is correlated with the expression of *FAD2* and *FAD3*, respectively [5, 33]. In addition, *FAD2* (*Atru.chr3.2406*) and *FAD3* (*Atru.chr3.4197*) were more highly expressed in the seeds at 115 DAF than at 85 DAF. Similarly, KCS is a rate-limiting enzyme mediating FA elongation, which is important for nervonic acid biosynthesis [2]. The data generated in the present study indicated the nervonic acid content in the seeds was 13.8-fold higher at 115 DAF than at 85 DAF. We previously identified 28 genes in the *A. truncatum KCS* gene family [2]. However, only *Atru.chr4.2308* was differentially expressed between the seeds at 85 and 115 DAF at the transcription, translation, and protein levels in this study. Accordingly, this gene may be important for nervonic acid biosynthesis. To know more the overall transcription and translation features of *FAD2* (*Atru.chr3.2406*), *FAD3* (*Atru.chr3.4197*), and *KCS* (*Atru.chr4.2308*). Figure S4 showed the three genes of RNA-seq and Ribo-seq coverage tracks by using Integrative Genomic Viewer (IGV). The results showed that the change tendency of mRNA and RPFs was similar in the seeds at 85 DAF and at 115 DAF, respectively. While the abundance of the seeds at 115 DAF was higher than that of 85 DAF. This may indicate that they are efficient in transcription and translation levels.

Transcription factors (TFs) have important roles related to post-transcriptional regulation. The regulatory effects of several TFs on lipid biosynthesis have been reported, including WRINKLED1 (WRI1) [34], leafy cotyledon1 (LEC1), fusca3 (FUS3), abscisic acid insensitive 3 (ABI3), v-myb avian myeloblastosis viral oncogene homolog (MYB), and DNA-binding with one finger (Dof) [5, 35–37]. However, the transcriptional regulation of lipid biosynthesis in *A. truncatum* seeds has not been comprehensively elucidated. In this study, 1,510 TFs were identified during the examination of the seeds collected at two time points (Table S10). *A. truncatum* is a versatile oil-producing woody tree and a rich source of oleic acid (C18:1), linoleic acid (C18:2), and nervonic acid (C24:1) [3–5]. To identify the TFs that directly regulate the expression of the oil biosynthesis pathway genes *FAD2* (*Atru.chr3.2406*), *FAD3* (*Atru.chr3.4197*), and *KCS* (*Atru.chr4.2308*), we completed a co-expression analysis to identify TF genes with expression patterns that were significantly and highly correlated (*r* > 0.7, *P* < 0.05) with the expression patterns of the three genes in the oil biosynthesis pathway (Fig. 5C). We detected 31, 51, and 37 TF genes that were co-expressed with *FAD2* (*Atru.chr3.2406*), *FAD3* (*Atru.chr3.4197*), and *KCS* (*Atru.chr4.2308*), respectively (Table S11). Additionally, the known MYB and ABI TFs affected the expression of *FAD2* (*Atru.chr3.2406*), whereas the bZIP, MYB, and Dof TFs modulated the *FAD3* (*Atru.chr3.4197*) and *KCS* (*Atru.chr4.2308*) expression levels. These findings may provide important clues regarding the molecular basis of lipid biosynthesis in *A. truncatum* seeds.

### Identification and analysis of small ORFs in UTRs

Untranslated regions are key mediators of post-transcriptional regulation. Moreover, CDS translation is modulated by the combined effects of uORFs, dORFs, the secondary structure around uORFs, and the distance between the uORF and CDS [36]. Earlier studies suggested that uORFs affect TE [38, 39]. However, it remains unknown whether the annotated uORFs in *A. truncatum* can influence gene transcription or translation. The ribosomal profile data generated in the current study will enable the identification and analysis of uORFs in *A. truncatum*. Information regarding the uORFs, dORFs, and mORFs identified by our analysis of *A. truncatum* seeds is provided in Table S12. The uORFs and dORFs were distinguished from the known protein-coding genes according to RRS and ORFscore data (Fig. S5A and B). A total of 2,151 uORFs were identified, which accounted for 9.67% of the transcripts.

**Fig. 6:**
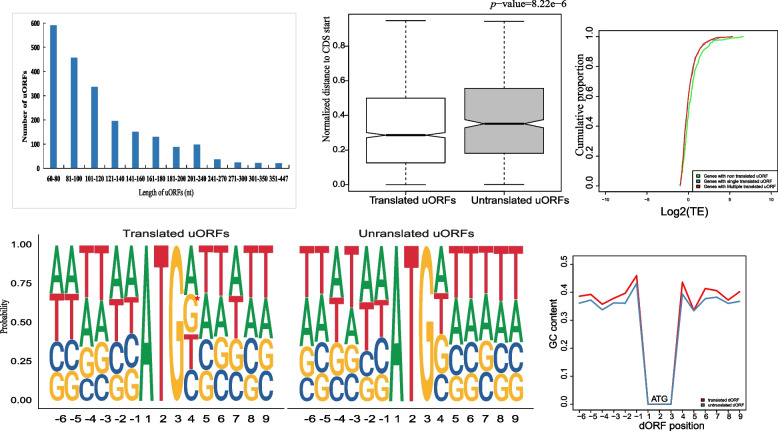
Features of uORFs in 85DAF *A. truncatum* seeds. **A** Length distribution of uORFs. **B** Normalized distance from uORFs to CDS ATG start codon (85DAF). **C** The relationship between the length of uORF and TE (85DAF). **D** Sequence composition between translated and untranslated uORFs around the ATG start codon. Chi-squared test was used for significance analysis. The asterisks indicate *P*-value < 0.05 (85DAF). **E** GC content of the flanking sequence of translated (red line) and untranslated (blue line) uORFs’ ATG start codons, respectively

Figure 6 presents the features of the uORFs detected in the seeds collected at 85 DAF. Most of the identified uORFs consisted of 60–460 nt, with 80% of the uORFs shorter than 160 nt (Fig. 6A). This observation was following the uORF length distribution in *A. thaliana*, tea, rice, and other higher plants [38, 40, 41]. Furthermore, we observed that the relative distance from uORF to the start codon of the mORFs (*P*-value = 8.22e − 6) in translated uORFs was shorter than non-translated uORFs (Fig. 6B). Of the genes with a relatively high TE (> 0.5), the cumulative curve showed that TE tended to be lower for the genes with a translated uORF than for the genes with a non-translated uORF. In contrast, there were no significant differences between the genes with a single translated uORF and the genes with multiple translated uORFs (Fig. 6C). Moreover, Kozak consensus sequence with “GCCA/GCCAUGG,” especially the position of − 3(A/G) and 4(G) around AUG start codon is important for start codon recognition as well as translation initiation [42]. We first performed motif analysis around the start codon of mORFs, the frequency of each base of the flanking sequence around the uORF start codon between translated and untranslated was analyzed, and a high GC content of the flanking sequence in translated uORFs was observed (Fig. 6D and E). Furthermore, the probabilities of guanine at the position of + 4 (*P*-value = 0.02) in translated uORFs were found to be higher than in untranslated ones (Fig. 6D). Similar results were obtained for the seeds collected at 115 DAF (Fig. S6).

## Discussion

*A. truncatum* is an important woody tree species that produce seeds with large amounts of unsaturated fatty acids [2]. Thus, the molecular mechanism underlying lipid synthesis in the key seed development stages must be characterized. Previous research determined that 85 and 115 DAF are crucial time points for FA synthesis during the *A. truncatum* seed development stage. Our comparative analyses of transcriptional and translational profiles of seeds collected at 85 and 115 DAF provide new insights into the global mechanisms underlying the developmental regulation of lipid metabolism.

Based on our analyses, we identified the expressed uORFs in *A. truncatum*. Translational efficiency is influenced by the presence of uORFs that repress translation, which is consistent with our finding that genes with translated uORFs have a lower TE than genes with non-translated uORFs [43]. Some researchers have speculated that long uORFs may compete for translation initiation factors, and the distance between expressed uORFs and the CDS affects TE in mammalian and yeast cells [43]. Wu et al. [11] reported that the uORF length is not highly correlated with TE. Our results suggested that the sequence flanking ATG was perfectly accorded with the Kozak sequence in mORFs, the repressive effects of uORFs on translation may be associated with their unique features in different species.

In oilseed plants, oil synthesis mainly involves de novo FA biosynthesis, TAG assembly, and oil body formation [4]. Although the genes in the main FA biosynthesis pathways have been identified [2, 5], the number of key genes and their expression patterns in developing seeds vary among species. In the current study, the oil synthesis pathway was constructed by combining the results of a multi-omics investigation. We also examined the key genes at the translatomic and proteomic levels. At the transcription, translation, and protein levels, *LACS* (*Atru.chr.9.2000*, *Atru.chr1.2506*, *Atru.chr6.342*), *FAD2* (*Atru.chr3.2406*), *FAD3* (*Atru.chr3.4197*), and *KCS* (*Atru.chr4.2308*) expression levels were highly correlated with FA contents. Hence, the expression of these genes, which encode key enzymes in the lipid biosynthesis pathway, is potentially regulated by post-translational modifications. Additionally, our findings are suggestive of the post-translational regulation of lipid biosynthesis by the identified TFs (e.g., MYB, ABI, bZIP, and Dof). Notably, lipid biosynthesis in plants is influenced by various factors, including the cultivar, tissue, growth stage, and environmental stimuli. In this study, we combined our analyses of phenotypic changes, differential expression, and co-expression with an examination of TE to reveal the molecular basis of *A. truncatum* seed oil production. However, future studies will need to precisely decipher the associated post-translational regulation in *A. truncatum*.

## Conclusions

We combined Ribo-seq and RNA-seq techniques, enabled the identification of ORFs in *A. truncatum* seeds and revealed their translational features. To the best of our knowledge, this is the first comprehensive multi-omics analysis of lipid metabolism.

We compared the analyses of transcriptional and translational profiles of seeds collected at 85 and 115 DAF. And the key genes and TFs were examined at the translatomic and proteomic levels. The features of uORFs in *A. truncatum* seeds also provide new insights into the global mechanisms underlying the developmental regulation of lipid metabolism. The results will serve as an important foundation for future explorations of the mechanisms regulating lipid accumulation in *A. truncatum* and oilseed crops.

## Methods

### Plant materials and sampling

*A. truncatum* plants were grown in the Jiangsu Province Aceraceae Germplasm Repository (Lishui district, Nanjing, China; latitude: 31°65′N, longitude: 119°02′E) under natural conditions (each has three biological replicates). Seeds were collected at 85 and 115 DAF from three individuals for the RNA-seq and Ribo-seq analyses, oil content measurements, and protein extraction and digestion. The collected samples were immediately frozen in liquid nitrogen and stored at − 80 °C until analyzed.

### Lipid analysis and oil body examination

Fifty seeds collected from three plants (each has three biological replicates) at 85 and 115 DAF were used for extracting oil and FA methyl esters as described by Gao et al. [44] and Ma et al. [5]. The FAs were analyzed using the TRACE DSQ gas chromatography–mass spectrometry system (Thermo Fisher Scientific Inc., Waltham, MA, USA) as described by Zhang et al. [45]. The embryos were cut in the middle and then immediately fixed in a solution comprising 4% (v/v) glutaraldehyde and sodium phosphate buffer (pH 7.2). They were subsequently post-fixed in osmium tetroxide, dehydrated, and embedded in Spurr’s resin. The sections were examined using the JEM-1400 transmission electron microscope (60 kV) (JEOL, Tokyo, Japan).

### RNA extraction, library construction, and sequencing

TRIzol reagent (Invitrogen, NJ, USA) was used to extract total RNA from the developing seeds collected from three plants at 85 and 115 DAF. The RNA-seq libraries were constructed using the NEBNext Ultra Directional RNA Library Prep Kit (New England BioLabs, Ltd., USA). Six cDNA libraries were sequenced on the NovaSeq 6000 platform (Illumina, San Diego, CA). After removing duplicate reads, adapters, and ambiguous sequences (i.e., reads with an N rate exceeding 10%), the remaining clean reads were mapped to our *A. truncatum* reference genome (the Sequence Read Archive (SRA) under study accession number SUB6287730) [2]. using HISAT2 (version 2.0.4) (parameters: -rna-strandness RF) [46]. Differentially expressed genes (DEGs) were analyzed using the DESeq2 software (FDR < 0.05 and absolute fold-change ≥ 1.5) [47]. Significant DEGs were identified based on the following criteria: false discovery rate (FDR) < 0.05 and∣log2fold-change∣ ≥ 1.5. The Gene Ontology (GO) (http://www.geneontology.org/) and Kyoto Encyclopedia of Genes and Genomes (KEGG) (http://www.genome.jp/kegg/) databases were used to functionally annotate genes and identify enriched metabolic pathways (R project package Rscript.3.6.0), respectively. To search for transcription factors (TFs) among DEGs, the data were screened against TF database PlantTFDB (http://planttfdb.gao-lab.org/).

### Ribosome footprints (RFs) extraction and Ribo-seq library construction

The RFs were extracted as below: the seeds frozen in liquid nitrogen were treated with 400 µL lysis buffer for 10 min. The lysate was centrifuged at 20,000 × g for 10 min at 4 °C, after which the supernatant was collected. To prepare the RFs, 10 μL RNase I (NEB, Ipswich, MA, USA) and 6 μL DNase I (New England BioLabs, Ltd., USA) were added to 400 μL lysate supernatant, which was incubated for 45 min at room temperature with gentle mixing on a Nutator mixer. The RNases in the samples were digested with 10 μL SUPERase in RNase Inhibitor (Ambion, Austin, TX, USA). The digested Ribosome Protected Fragments (RPFs) were loaded onto the Illustra MicroSpin S-400 HR column and centrifuged at 600 × g for 3 min. Next, 10 μL 10% (w/v) SDS was added to the eluted samples, then RFs longer than 17 nt were isolated using the RNA Clean and Concentrator-25 kit (Zymo Research). To eliminate rRNA, antisense DNA probes (50–80 bases) complementary to rRNA sequences were added to the RPFs, after which RNase H (New England BioLabs, Ltd., USA) and DNase I (New England BioLabs, Ltd., USA) were added to digest the rRNA and residual DNA probes. Finally, RFs were further purified using magnetic beads (Vazyme, Nanjing, Jiangsu, China) [48]. Additionally, Ribo-seq libraries were constructed using the NEBNext® Multiple Small RNA Library Prep Set for Illumina® [49] and then sequenced on the Illumina HiSeq™ X10 system by Gene Denovo Biotechnology Co. (Guangzhou, China).

### Ribosome profiling raw data processing and analysis

The raw data were filtered to remove reads containing adapters, poly-N sequences, and more than 50% low-quality bases using an in-house Perl script. After mapping the retained reads to the sequences in the ribosomal RNA (rRNA), GenBank, and Rfam databases using Bowtie2 (version 2.2.8) (parameters:-p 4 -k 10 –no-unal –phred64), the reads mapped to rRNAs, transfer RNAs (tRNAs), small nuclear RNAs (snRNAs), small nucleolar RNAs (snoRNAs), and miRNAs were eliminated [50]. Next, the processed reads were mapped to our *A. truncatum* reference genome (SUB6287730) [2] using STAR (version 3.1.0) (ThreadN 4 –runMode alignReads –readFilesCommand zcat –twopassMode Basic), with no mismatches allowed. Read counts in the ORFs of protein-coding genes were calculated using the RSEM software (version 1.2.19) (est_method RSEM –aln_method bowtie2 –seqType fq –thread_count 4 –seed-length 10) and the gene expression level was normalized by using FPKM (fragments per kilobase of transcript per million mapped reads) values. The DEGs (FDR < 0.05 and absolute fold-change ≥ 1.5) were functionally annotated and enriched metabolic pathways were identified according to the GO and KEGG databases, respectively. The three-nucleotide periodicity was plotted using the riboWaltz R package [50], whereas the potential ribosomal pause sites (single-codon resolution) were investigated using PausePred (https://pausepred.ucc.ie/).

### Identification and quantitative analysis of ORFs

A custom ORFfinder search was conducted using transcript sequences annotated as non-coding regions, including 5′ and 3′ UTRs. Noncanonical ORFs, including uORFs and downstream ORFs (dORFs), were extracted using start codons (e.g., ATG). The raw reads containing adapters as well as reads containing poly-N sequences and over 50% low-quality bases were removed using an in-house Perl script. The reads mapped to rRNA and tRNA database sequences were eliminated using Bowtie2. The training reads were mapped to our *A. truncatum* reference genome (SUB6287730) [2] using Bowtie2, with no mismatches allowed. The reads mapped to snRNAs, snoRNAs, and microRNA precursor regions were not considered further. Finally, the main ORFs (mORFs) of the protein-coding genes as well as the uORFs and dORFs were used to analyze the translation level. The DESeq2 package (http://www.r-project.org/) was used for identifying differentially translated ORFs (FDR < 0.05 and absolute fold-change ≥ 1.5). The ribosome release score (RRS) and ORFscore were used to evaluate the coding potential of non-canonical ORFs [51]. The 95th percentiles of the RRS and ORFscore data as well as the Fickett and hexamer scores calculated using the CPAT tool [52] were used to assess the coding potential of different ORFs.

### DIA-based protein quantification

Proteins were extracted from seeds and digested as described by Wu et al. [11]. Briefly, 2 mL lysis buffer (Roche Ltd., Basel, Switzerland) was added to the seeds frozen in liquid nitrogen. The solution was sonicated (on ice for 30 min) and then centrifuged (13,000 rpm for 30 min at 4 °C). The proteins in the supernatant were precipitated with ice-cold acetone during an overnight incubation at − 20 °C. Protein quality was evaluated by sodium dodecyl sulfate–polyacrylamide gel electrophoresis. The extracted protein (50 μg) was suspended and incubated. The obtained proteins were digested with modified trypsin (Promega Co, Ltd., Madison, USA) at a substrate: enzyme ratio of 50:1 (w/w). The peptide mixture was dissolved and fractionated using the Ultimate 3000 system (Thermo Fisher Scientific, MA, USA). The peptides were redissolved and analyzed by LC–MS/MS. Specifically, the Orbitrap Fusion Lumos mass spectrometer coupled to the EASY-nLC 1200 system (Thermo Fisher Scientific) was used. The raw DIA data were processed and quantified using the default parameters of Spectronaut Pulsar 11.0 (Biognosys AG, Zurich, Switzerland). The differentially regulated proteins (DRPs) were filtered (fold-change ≥ 1.5 or < 0.67; *P* < 0.05) [53]. The identified proteins were annotated using the GO and KEGG databases.

### Quantitative real-time PCR (qRT-PCR)

The expression levels of the DEGs related to FA biosynthesis were validated by qRT-PCR analysis. Specific primers were designed using Primer5 (Table S1). Total RNA was extracted as previously described. Next, 1 μg RNA served as the template for the synthesis of first-strand cDNA using the PrimeScript Reagent Kit (TaKaRa, Dalian, China). The cDNA templates were diluted 20-fold before use. The qRT-PCR analysis was performed using SYBR Premix ExTaq (TaKaRa Bio, Kusatsu, Japan) and the StepOne Plus Real-Time PCR System (Applied Biosystems, Thermo Fisher Scientific). *Actin* was selected as the internal reference gene [54]. Gene expression levels were analyzed according to the 2^−ΔΔCt^ method [55].

## Supplementary Information

**Additional file 1: Fig. S1.** Principal component analysis on the RNA-seq (A) and Ribo-seq (B) and proteomic profiles (C) of three replicates of 85 and 115 DAF seeds in *A. truncatum*. **Fig. S2.** The GC-MS analysis of 85 DAF and 115 DAF seeds in *A.truncatum*. **Fig. S3.** (A): DEGs of translational efficiency in 115 DAF vs. 85 DAF. (B): KEGG pathway enrichment analysis of DEGs of translational efficiency in 115 DAF vs. 85 DAF. **Fig. S4.** The RNA-seq and Ribo-seq coverage tracks of *FAD2* (*Atru.chr3.2406*), *FAD3*(*Atru.chr3.4197*) and *KCS* (*Atru.chr4.2308*) by using Integrative Genomic Viewer (IGV). **Fig. S5. **The scatterplots of log_10_(Ribosome release score) against ORF score of ORFs (A) and Fickett score against Hexamer score. (B) The dasher lines represent the 95^th^ percentiles set as threshold values. **Fig. S6. **Features of uORFs in 115DAF *A. truncatum* seeds.

**Additional file 2: Supplementary Table S1.** Primers (5´-3´) used in the quantitative real-time polymerase chain rection (qRT- PCR) to validate the reliability of the RNA-seq results.

**Additional file 3: Supplementary Table S2.** Statistic on the RNA-seq data and ribosomal profiling data.

**Additional file 4: Supplementary Table S3.** The identification and quantification information of transcriptome.

**Additional file 5: Supplementary Table S4.** The identification and quantification information of translatome.

**Additional file 6: Supplementary Table S5.** The identification and quantification information of proteome.

**Additional file 7: Supplementary Table S6.** The gene information in different quadrants at transcription and translation levels.

**Additional file 8: Supplementary Table S7.** The gene information in different quadrants at the transcript and protein levels.

**Additional file 9: Supplementary Table S8.** The contents of fatty acids in 85 DAF and 115 DAF *A. truncatum* seeds.

**Additional file 10: Supplementary Table S9.** The KEGG analysis of differential translational efficiency genes in 85 DAF and 115 DAF *A. truncatum* seeds.

**Additional file 11: Supplementary Table S10.** The information of identified TFs in 85 DAF and 115 DAF *A. truncatum* seeds.

**Additional file 12: Supplementary Table S11. **The information of identified TF genes were co-expressed with *FAD2 *(*Atru.chr3.2406*), *FAD3 *(*Atru.chr3.4197*), and *KCS *(*Atru.chr4.2308*).

**Additional file 13: Supplementary Table S12. **The information of uORFs and dORFs identified as potential coding sequences based on RRS and ORF score.

## Data Availability

The RNA-seq raw data (accession number: SUB12016538) and Ribo-seq raw data (accession number: SUB12025717) were uploaded to National Center for Biotechnology Information under Project No. PRJNA877423 [56]. The mass spectrometry proteomics data (Project No. IPX0005014000) was uploaded to the iProX integrated proteome resources.

## Abbreviations

RPFs: Ribosome-protected fragments
uORFs: Upstream open reading frames
dORFs: Downstream open reading frames
5′UTR: 5′ upstream untranslated region
CDS: Protein-coding sequences
DEG: Differentially expressed genes
TE: Translational efficiency
PDHC: Pyruvate dehydrogenase complex
ACCase: Acetyl-CoA carboxylase
MCMT: Malonyl-CoA-ACP transacylase
KASI/II/III: Ketoacyl-ACP synthase I/II/III
KAR: Ketoacyl-ACP reductase
HAD: Hydroxyacyl-ACP dehydrase
EAR: Enoyl-ACP reductase
SAD: Stearoyl-ACP desaturase
FAD2/6: Oleate desaturase
FAD3/7/8: Linoleate desaturase
FATA/B: Acyl-ACP thioesterase A/B
PCH: Palmitoyl-CoA hydrolase
LACS: Long-chain acyl-CoA synthetase
LPCAT: Lysophosphatidylcholine acyltransferase
GK: Glycerol kinase
GPAT: Glycerol-3-phosphate acyltransferase
LPAAT: 1-Acylglycerol-3-phosphate acyltransferase
PAP: Phosphatidic acid phosphatase
DGAT: Diacylglycerol acyltransferase
PDAT: Phospholipid:diacylglycerol acyltransferase
WRI1: WRINKLED1
LEC1: Leafy cotyledon1
FUS3: fusca3
ABI3: Abscisic acid insensitive 3
MYB: v-myb avian myeloblastosis viral oncogene homolog
Dof: DNA-binding with one finger
