# Supplementary material for: Ribosome footprint profiling enables elucidating the systemic regulation of fatty acid accumulation in Acer truncatum
Source: BMC Biol. 2023 Apr 3;21:68. doi: 10.1186/s12915-023-01564-8 (PMC10071632; doi:10.1186/s12915-023-01564-8)
Supplement: Supplementary file 1 — Additional file 1: Fig. S1. Principal component analysis on the RNA-seq (A) and Ribo-seq (B) and proteomic profiles (C) of three replicates of 85 and 115 DAF seeds in A. truncatum. Fig. S2. The GC-MS analysis of 85 DAF and 115 DAF seeds in A.truncatum. Fig. S3. (A): DEGs of translational efficiency in 115 DAF vs. 85 DAF. (B): KEGG pathway enrichment analysis of DEGs of translational efficiency in 115 DAF vs. 85 DAF. Fig. S4. The RNA-seq and Ribo-seq coverage tracks of FAD2 (Atru.chr3.2406), FAD3(Atru.chr3.4197) and KCS (Atru.chr4.2308) by using Integrative Genomic Viewer (IGV). Fig. S5. The scatterplots of log10(Ribosome release score) against ORF score of ORFs (A) and Fickett score against Hexamer score. (B) The dasher lines represent the 95th percentiles set as threshold values. Fig. S6. Features of uORFs in 115DAF A. truncatum seeds. [file 12915_2023_1564_MOESM1_ESM.docx]

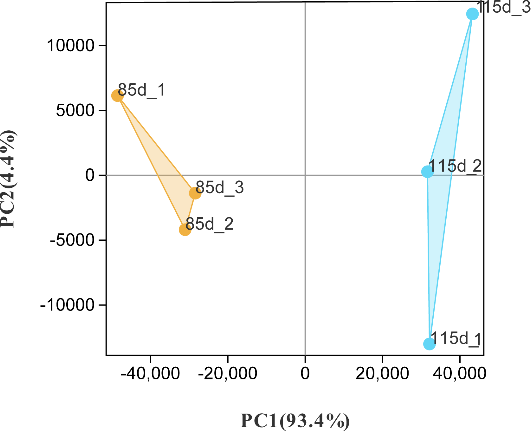


**A**


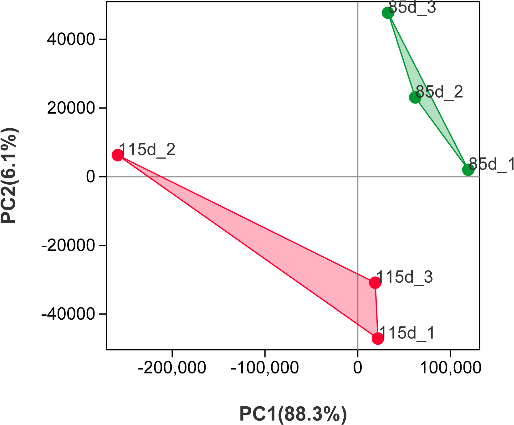


**B**


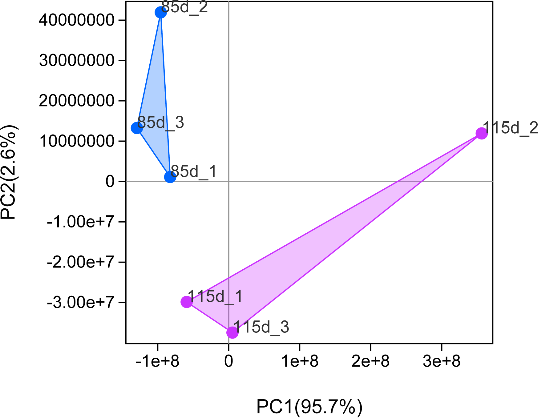


**C**

**Fig. S1.** Principal component analysis on the RNA-seq (A) and Ribo-seq (B) and proteomic profiles (C) of three replicates of 85 and 115 DAF seeds in *A. truncatum*.


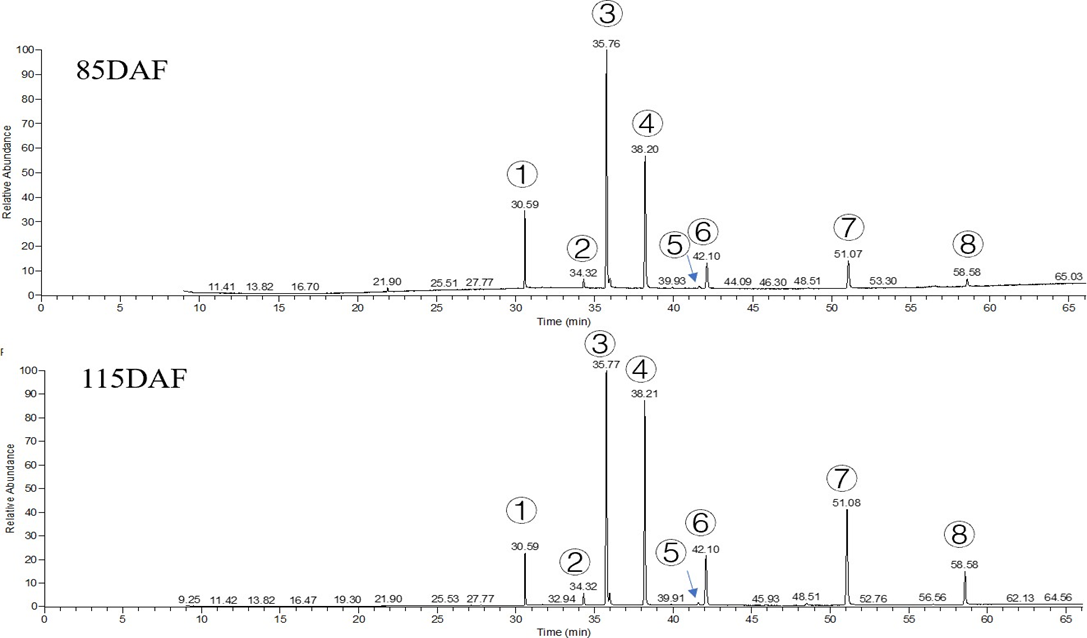


**Fig. S2.** The GC-MS analysis of 85 DAF and 115 DAF seeds in *A.truncatum*①Palmitic acid ②Stearic acid ③Oleic acid ④Linoleic acid ⑤Linolenic acid ⑥Eicosenoic acid ⑦Erucic acid ⑧Nervonic acid.


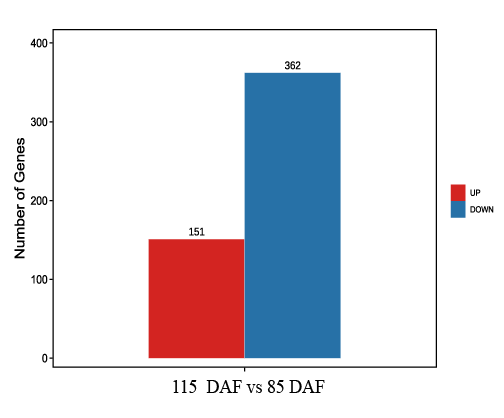

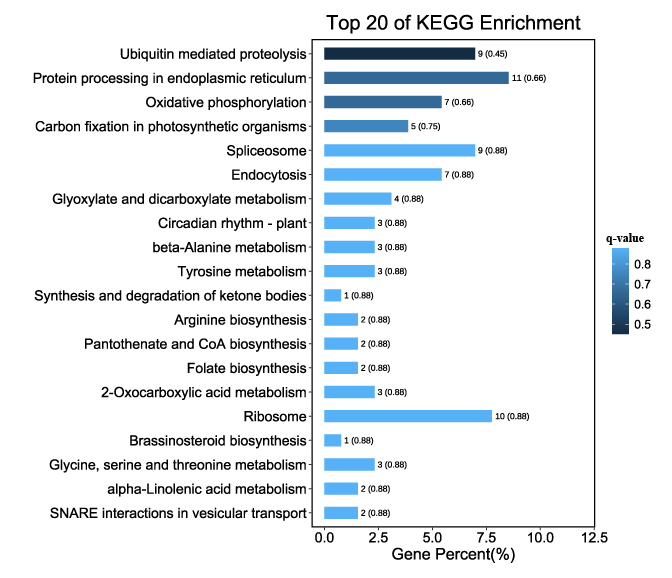


B

A

**Fig. S3.** **(A):** DEGs of translational efficiency in 115 DAF vs. 85 DAF. (B): KEGG pathway enrichment analysis of DEGs of translational efficiency in 115 DAF vs. 85 DAF.





**Fig. S4.** The RNA-seq and Ribo-seq coverage tracks of *FAD2* (*Atru.chr3.2406*), *FAD3*

(*Atru.chr3.4197*) and *KCS* (*Atru.chr4.2308*) by using Integrative Genomic Viewer (IGV).


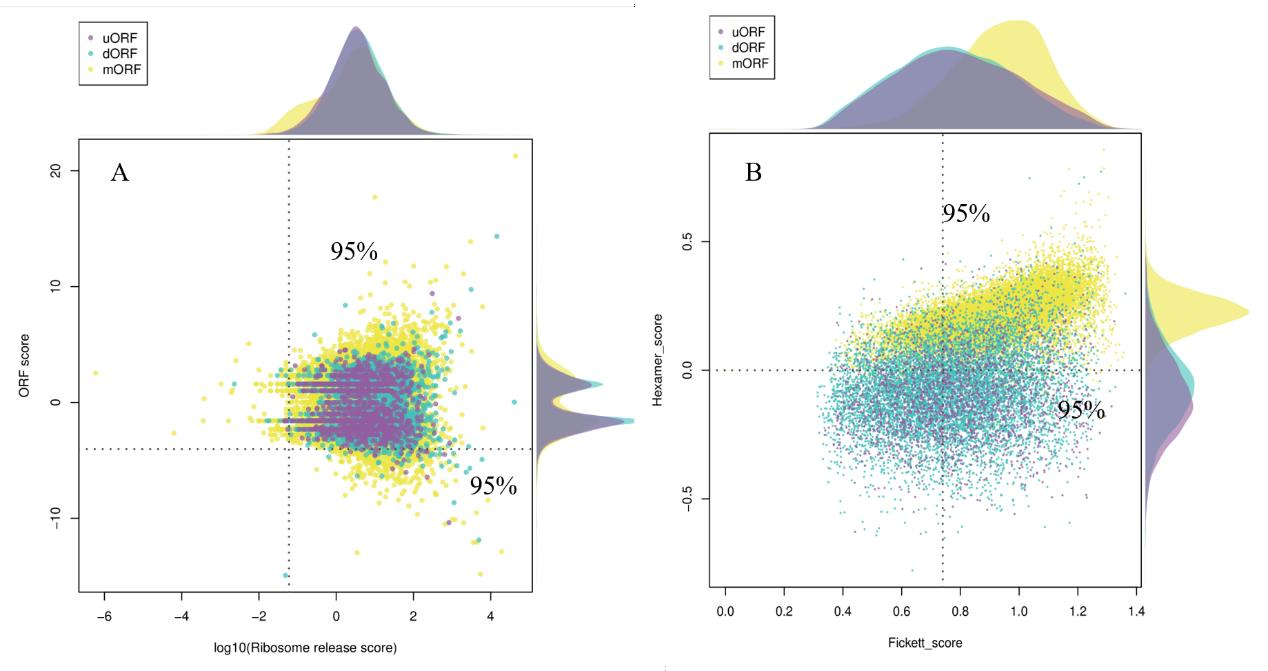


**Fig. S5.** The scatterplots of log10(Ribosome release score) against ORF score of ORFs (A) and Fickett score against Hexamer score. (B) The dasher lines represent the 95th percentiles set as threshold values.


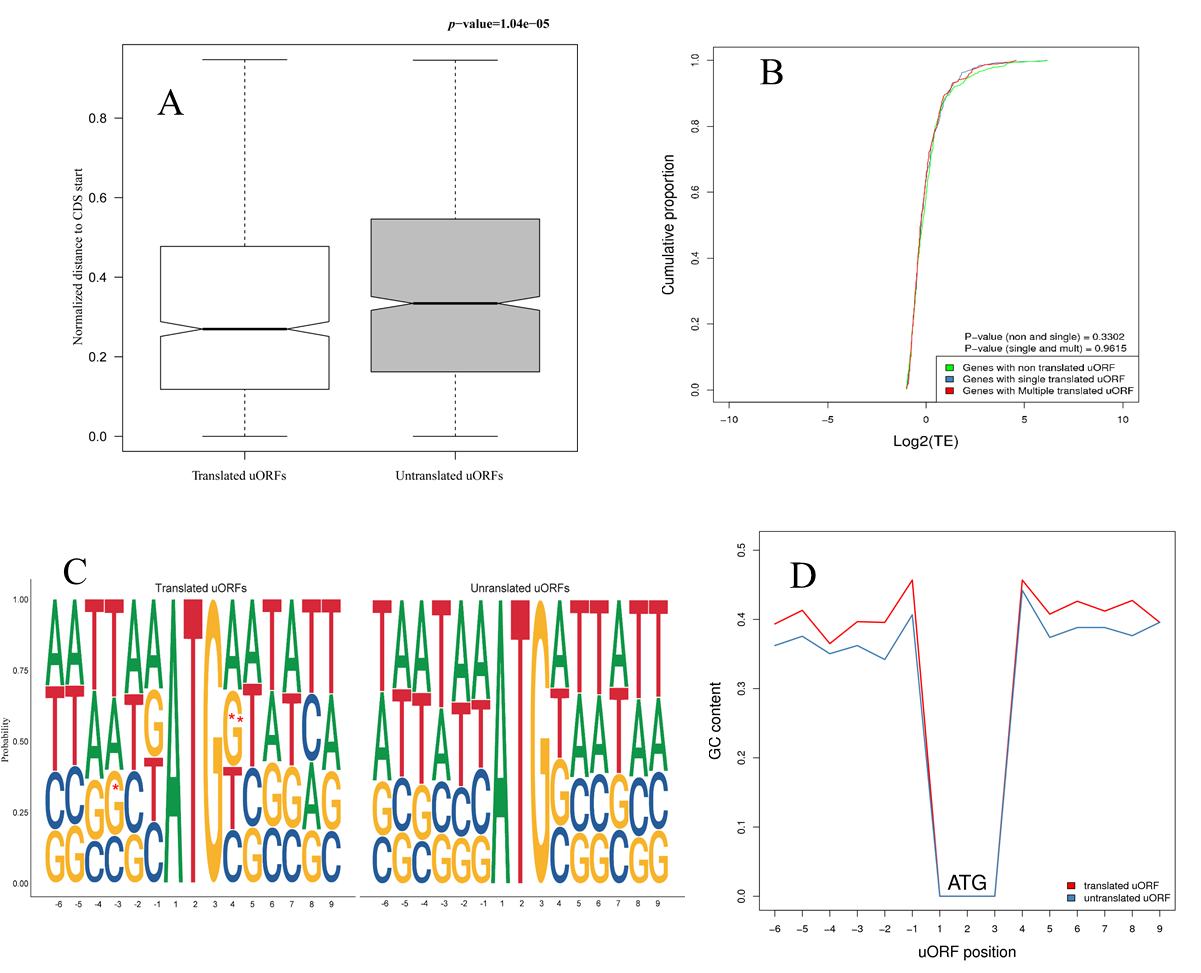


**Fig. S6.** Features of uORFs in 115DAF *A. truncatum* seeds. (A)normalized distance from uORFs to CDS ATG start codon (85DAF). (B) The relationship between the length of uORF and TE (85DAF). (C) Sequence composition between translated and untranslated uORFs around the ATG start codon. Chi-squared test was used for signifificance analysis. Single and double asterisks indicate P-value <0.05 and P-value <0.01 respectively. (85DAF). (D)GC content of flanking sequence of translated (red line) and untranslated (blue line) uORFs’ ATG start codons, respectively.
